# Development of Inducible CD19-CAR T Cells with a Tet-On System for Controlled Activity and Enhanced Clinical Safety

**DOI:** 10.3390/ijms19113455

**Published:** 2018-11-03

**Authors:** Xingjian Gu, Dongyang He, Caixin Li, Hua Wang, Guanghua Yang

**Affiliations:** 1 International Research Center for Marine Biosciences at Shanghai Ocean University, Ministry of Science and Technology, Shanghai 201306, China; xingjian.gu@mavs.uta.edu; 2 National Pathogen Collection Center for Aquatic Animals, Shanghai 201306, China; 3 Shanghai Collaborative Innovation for Aquatic Animal Genetics and Breeding, Shanghai 201306, China; 4 Shanghai Telebio Biomedical Co., Ltd., Shanghai 201321, China; hdy_telebio@163.com (D.H.); cx-l824@163.com (C.L.); vector.telebio@163.com (H.W.)

**Keywords:** CAR, Tet-On, inducible vector, CD19, doxycycline

## Abstract

The tetracycline regulatory system has been widely used to control the transgene expression. With this powerful tool, it might be possible to effectively control the functional activity of chimeric antigen receptor (CAR) T cells and manage the severe side effects after infusion. In this study, we developed novel inducible CD19CAR (iCAR19) T cells by incorporating a one-vector Tet-on system into the CD19CAR construct. The iCAR19 T cells showed dox-dependent cell proliferation, cytokine production, CAR expression, and strong CD19-specific cytotoxicity. After 48 h of dox induction, the relative CAR expression of induced cells was five times greater than that of uninduced cells. Twenty-four hours after dox removal, CAR expression significantly decreased by more than 60%. In cytotoxicity assays, dox-treated cells induced significantly higher specific lysis against target cells. These results suggested that the activity of iCAR19 T cells was successfully controlled by our Tet-on system, offering an enhanced safety profile while maintaining a robust anti-tumor effect. Besides, all manufacture processes of the lentiviral vectors and the T cells were conducted according to the Good Manufacturing Practice (GMP) standards for subsequent clinical translation.

## 1. Introduction

Chimeric antigen receptor T-cell therapy has shown great potential fighting against cancer, which functions by re-directing T cells to the tumor-specific antigen through genetic modification. Among different CAR T therapies, CAR T cells targeting the CD19 antigen has undergone extensive research and demonstrated remarkable therapeutic effects in clinical treatment for relapsed or refractory B-cell malignancies [1,2,3].

Despite the encouraging clinical success achieved in recent years, the potentially dangerous side effects associated with CAR T therapies remain a major concern in its clinical application, such as cytokine release syndrome (CRS), neurotoxicity, and low blood cell counts [4,5,6]. To minimize these side effects, efforts have been made in searching for adjuvant drugs able to control severe CRS [1,7], and novel CAR constructs incorporating suicide gene systems which induce CAR T-cell apoptosis upon activation [8,9,10]. However, these approaches might have some limitations. While adjuvant drugs increase the financial burden to families and societies, these chemicals could also induce a certain level of toxicity themselves. In terms of the suicide gene systems like the inducible caspase 9, direct elimination of infused CAR T cells may result in an increased risk of disease recurrence.

To overcome these potential limitations, the tetracycline regulatory system might be an ideal alternative since the transgene expression can be regulated by a very small dose of doxycycline (Dox) without the need to kill the CAR T cells. In typical Tet-on systems, a reverse tetracycline-controlled trans-activator (rtTA) can bind with the tetracycline-responsive element (TRE) promoter in the presence of doxycycline and activates transcription [11,12].

In the present study, we introduced an all-in-one Tet-on system into the third-generation lentiviral vector containing a CD19CAR construct, to explore the feasibility of regulating CD19CAR T-cell activity by doxycycline. For higher induction efficiency and lower expression leakage, the rtTA2S-M2, which is a mutant derived from rtTA, and the Tet-responsive promoter for albumin (TREalb) were used in the Tet-on system, as described in our previous publication [13]. In addition, to produce GMP-grade lentiviral vectors, all the manufacture processes and subsequent testing were carried out in our GMP facility, and the quality control of the gene vectors was performed following the FDA guidance.

## 2. Results

### 2.1. Construction of Inducible Lentiviral Vectors

**Figure 1:**
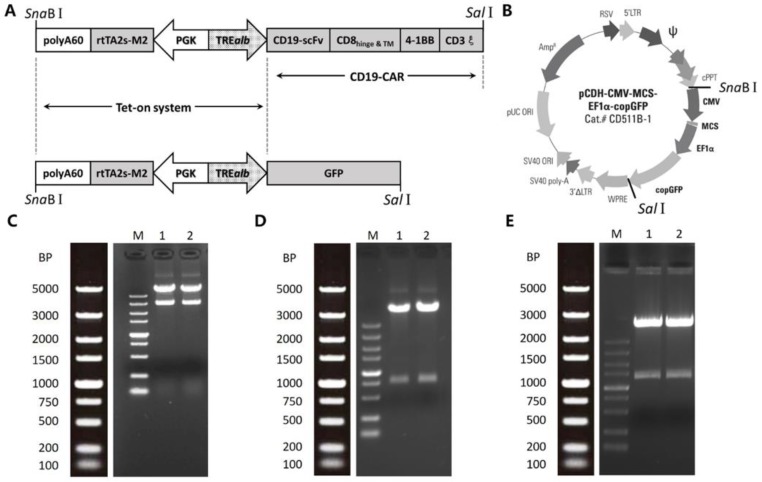
Vector construction and restriction endonuclease analysis. (**A**) The iCAR19 construct (upper) consisted of the Tet-on sequence and the CD19-CAR fragment. The iGFP construct (lower) consisted of the Tet-on sequence and the GFP sequence. (**B**) Constructs in (**A**) were respectively cloned into the pCDH-CMV-MCS-EF1α-copGFP lentiviral backbone between SnaBI and SalI sites. (**C**−**E**) the restriction endonuclease analysis results of pCDH-induce (**C**), iGFP (**D**), and iCAR19 (**E**) transfer vector plasmid. SnaBI-SalI digestion was used for pCDH-induce vector. SalI digestion was used for iGFP and iCAR19 vectors.

As shown in Figure 1A, the one-vector Tet-on sequence was composed of the rtTA2s-M2 cassette and the TREalb cassette, oriented in the antisense direction, as described in Reference [13]. The iCAR19 construct consisted of the Tet-on sequence and the CD19CAR fragment. The CD19CAR consisted of the CD19-specific single-chain variable fragment (scFv) derived from the murine FMC63 monoclonal antibody, in frame to CD8 hinge and transmembrane domains, 4-1BB transactivation domain, and CD3 zeta signaling domain, as previously reported [14]. The iGFP construct consisting of the Tet-on sequence and the GFP sequence was designed as a control. The two constructs were synthesized and cloned into the pCDH-CMV-MCS-EF1α-copGFP lentiviral backbone between SnaBI and SalI sites, respectively, to generate the transfer vectors. For restriction endonuclease analysis (Figure 1C−E), SnaBI-SalI digestion was performed for pCDH-induce vector, and SalI digestion was performed for iGFP and iCAR19 vectors. All vectors were correctly constructed.

### 2.2. Quality Testing

To ensure the quality of the iCAR19 lentiviral vector, a series of quality testing experiments were conducted (Table 1). For sterility, no bacteria, fungi or mycoplasma was detected in the vector stock. The infectious titer was determined as 1.5 × 10^7^ TU/mL by qPCR. For RCR test, no replication-competent lentivirus was detected in the cell culture supernatants after serial passages. The endotoxin presented in the stock was below 0.03125 EU/mL. The pH value of the stock was 7.25. In conclusion, the quality of our lentiviral vector complied with all criteria of the standard.

### 2.3. Dox-Induced GFP Expression in HEK293T Cells

**Figure 2:**
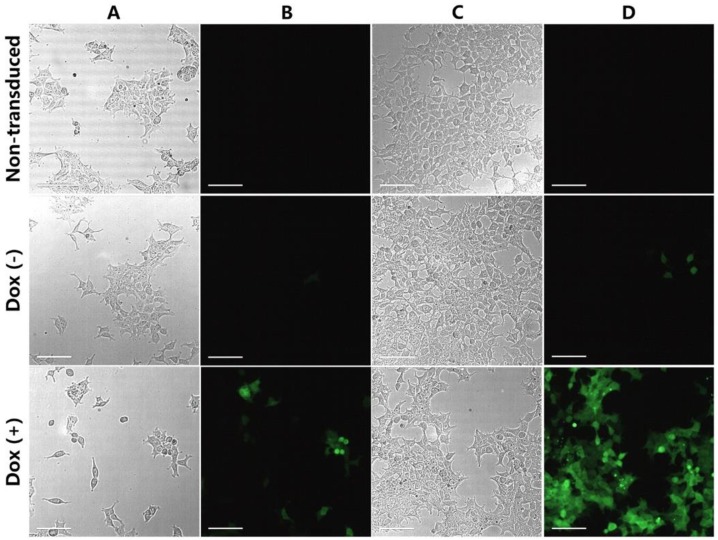
Dox-induced GFP expression in HEK293T cells. Dox (−), transduced PBMCs without Dox treatment. Dox (+), transduced PBMCs treated with Dox for 48 h. 24 h after induction (**A**,**B**), the Dox (+) group showed low level of GFP expression, and no GFP expression was observed in the non-transduced and Dox-groups. After 48 h of induction (**C**,**D**), most of the cells in the Dox (+) group showed high level of GFP expression, while only a small number of cells in the Dox (−) group showed GFP expression. Presented is the representative data. Scale bars indicate 50 μm.

HEK293T cells were transduced with the iGFP lentiviral vector to test the efficacy of our Tet-on system. Non-transduced cells were used as control. After 24 h of culture, 4 μg/mL doxycycline was added for the Dox (+) group. All three groups were maintained in culture for another 48 h, and GFP expression was examined 24 h and 48 h following doxycycline addition. As shown in Figure 2A,B, after 24 h of induction, some of the cells in the Dox (+) group already showed a low level of GFP expression, while almost no GFP expression was observed in the non-transduced and the Dox (−) groups. After 48 h of induction (Figure 2C,D), most of the cells in the Dox (+) group showed high level of GFP expression, whereas only a small number of cells in the Dox (−) group expressed GFP marker with a relatively low intensity. These results suggested that our one-vector Tet-on system effectively regulated the expression of the gene of interest.

### 2.4. Dox-Dependent CAR Expression in CAR T Cells

**Figure 3:**
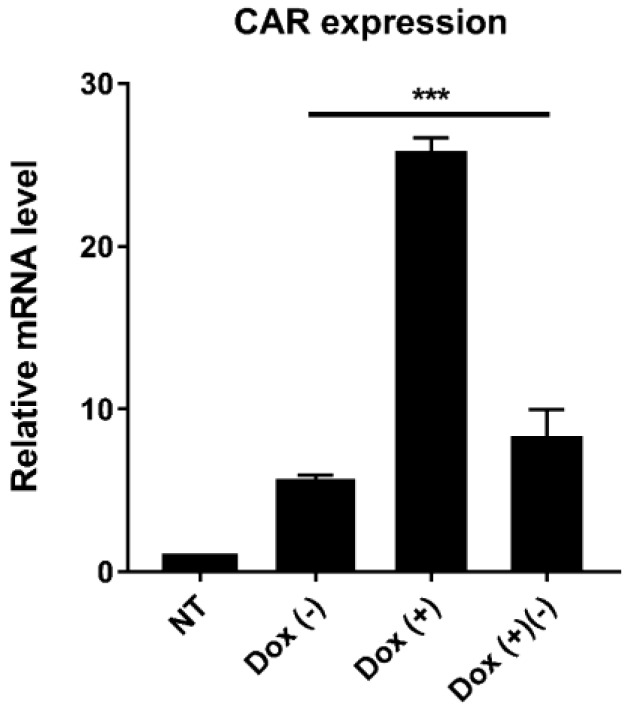
Quantitative real-time PCR analysis for dox-dependent CAR expression after 48 h of induction. NT, non-transduced PBMCs. Dox (−), transduced PBMCs without dox treatment. Dox (+), transduced PBMCs treated with dox for 48 h. Dox (+) (−), dox discontinuation of Dox (+) cells for 24 h. The CAR expression of Dox (+) was significantly higher than Dox (−) and Dox (+) (−). (Mean and SD, *n* = 3; *** *p* < 0.001).

To test whether the CAR expression can be regulated by dox administration and discontinuation, iCAR19 T cells were treated with or without 4 μg/mL doxycycline for 48 h. Subsequently, the dox-treated cells were washed and cultured without dox for another 24 h. The CAR expression in different groups was then compared on mRNA level by qPCR analysis (Figure 3). We observed a significant upregulation of CAR expression after dox induction. It was shown that the relative CAR expression of the induced group (25.9 ± 0.5) was significantly higher than the uninduced group (5.5 ± 0.3) when normalized to the non-transduced group. After removal of dox for 24 h, the CAR expression of dox-treated cells was significantly downregulated for more than 60% (8.3 ± 0.9). Besides, expression leakage of iCAR19 T cells was observed in the uninduced state by comparing the uninduced group with the control group.

### 2.5. Cell Proliferation and Cytokine Secretion

**Figure 4:**
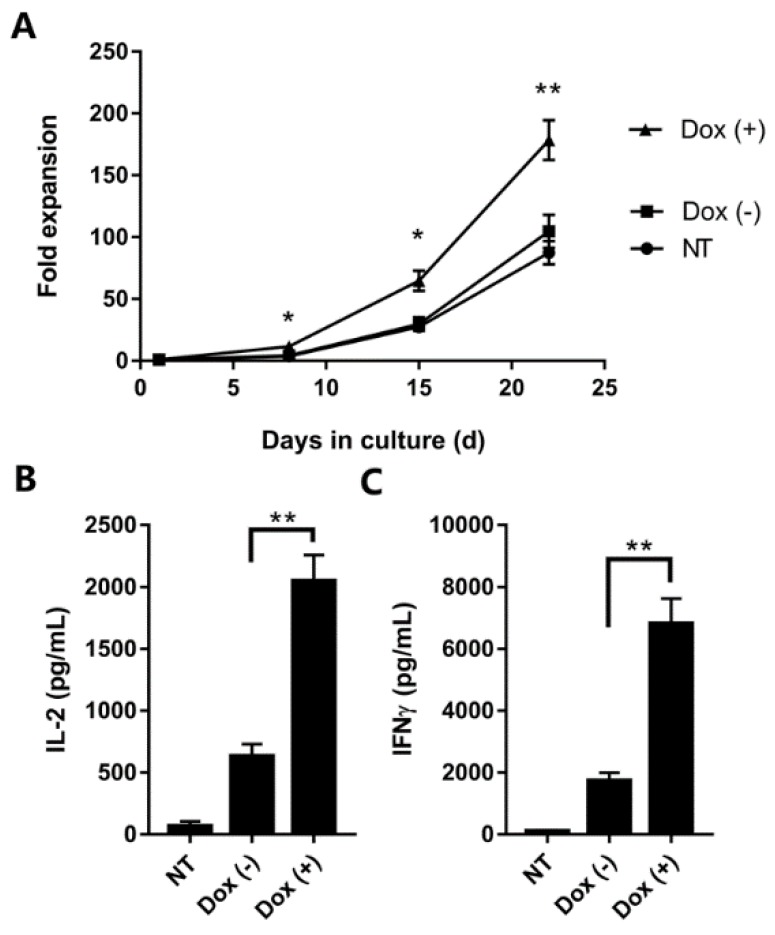
iCAR19 T-cell proliferation and cytokine secretion after CD19 stimulation were regulated by doxycycline administration. NT, non-transduced PBMCs. Dox (−), transduced PBMCs without Dox treatment. Dox (+), transduced PBMCs treated with Dox treatment. (**A**) Cell proliferation kinetics during 3 weeks of coculture with irradiated CD19^+^ LCLs. Cells were activated with CD3/CD28 beads, transduced and expanded in the IL-2 supplemented medium. (Mean and SD, *n* = 3; * *p* < 0.05; ** *p* < 0.01). (**B**,**C**) cytokine levels in supernatants after 24 h of coculture with irradiated Raji cells. Dox-treated groups showed significantly higher cell proliferation and cytokine induction. (Mean and SD, *n* = 3; ** *p* < 0.01).

Rapid expansion upon antigen stimulation is important for the anti-tumor activity of CAR T cells. To assess the ability of cell proliferation, iCAR19 T cells were activated with CD3/CD28 beads, transduced, and co-cultured with irradiated CD19^+^ LCLs in the IL-2 supplemented medium with or without doxycycline. Non-transduced PBMCs were used as control. During three weeks of coculture, viable cells were counted at weekly intervals. All groups demonstrated robust proliferation capability with more than 50-fold increase of total cell numbers (Figure 4A). Specifically, the fold expansion of induced cells was significantly higher than the control at all time points. There was significant difference in cell expansion between the induced and the uninduced group after day 15. No statistically significant difference was observed between the control and the uninduced group until day 22. We also examined the effect of dox administration on cytokine production of iCAR19 T cells. After 24 h of coculture with irradiated target cells, both induced and uninduced iCAR19 T cells yielded significant increase in IL-2 and IFNγ secretion in comparison to non-transduced cells (Figure 4B,C). Consistently, dox-induced iCAR19 T cells showed significantly higher cytokine production compared to the uninduced cells. These results suggest that cell proliferation and cytokine production of iCAR19 T cells were effectively regulated by the Tet-on system.

### 2.6. Cytotoxicity Assays

**Figure 5:**
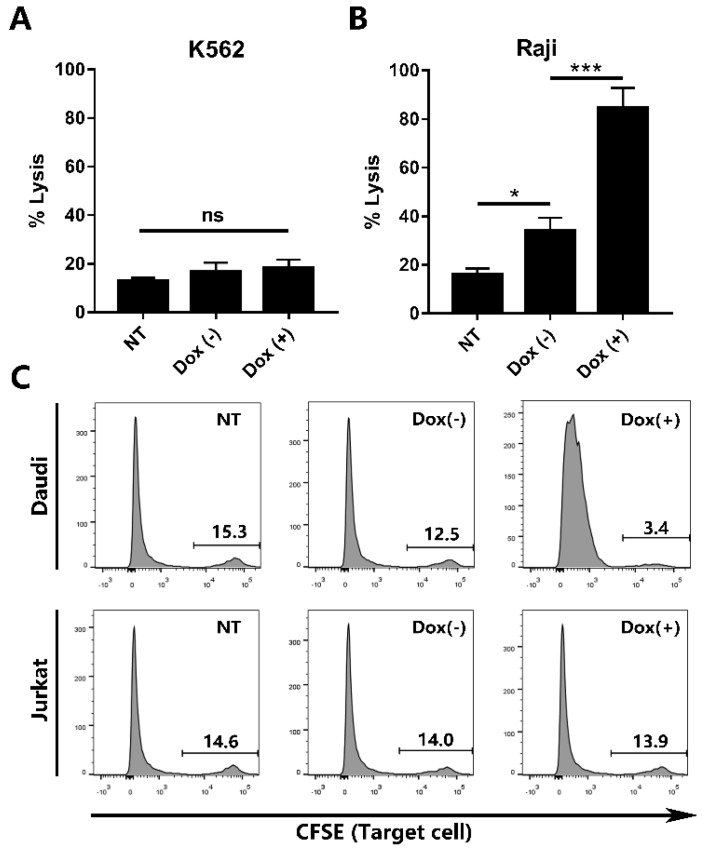
CAR T cells mediated dox-dependent and CD19-specific cytotoxicity. NT, non-transduced PBMCs. Dox (−), transduced PBMCs without Dox treatment. Dox (+), transduced PBMCs treated with Dox for 48 h. (**A**,**B**) bioluminescent-based cytotoxicity assays against K562 and Raji cell lines (Mean and SD, *n* = 3; * *p* < 0.05; *** *p* < 0.001; ns, not statistically significant). (**C**) Flow cytometry-based cytotoxicity assays against Daudi and Jurkat cell lines. Data are representative of three independent experiments.

The CD19-specific cytotoxicity of iCAR19 T cells was evaluated by the bioluminescent-based cytotoxicity assay using tumor cell lines expressing luciferase (Figure 5A,B). The uninduced and induced iCAR19 T cells were incubated with Raji or K562 cells at an E:T ratio of 5:1, and the non-transduced PBMCs served as control. After 16 h of co-incubation, Dox (+) cells induced significantly higher cytotoxic activity (84% of lysis) than Dox (−) cells (34% of lysis) against Raji cells, indicating a dox-dependent activity. Notably, the difference of cytotoxic activity to Raji cells between Dox (−) cells and non-transduced cells (16% of lysis) was also statistically significant, which indicated that a moderate level of functional leakage existed in this inducible system. This result was consistent with the previous fluorescence images and qPCR data (Figure 2 and Figure 3). While iCAR19 T cells exhibited strong cytotoxicity against Raji cells, they showed much lower cytotoxicity against CD19-negative K562 cells (less than 20% of lysis) with no statistical significance between each group, suggesting their CD19-specific cytotoxicity.

Additionally, flow cytometry-based cytotoxicity assay (Figure 5C) was performed against Daudi and Jurkat cells. The uninduced and induced iCAR19 T cells were co-cultured with CFSE-labeled target cells overnight at an E:T ratio of 5:1. The percentage of viable target cells were determined by flow cytometry. The percentage of Daudi cells largely decreased after co-culture with Dox (+) cells (3.9 ± 0.4, *n* = 3), whereas only a slight decline was observed for Dox (−) cells (11.4 ± 0.6, *n* = 3). The percentages of survival Daudi cells were statistically significant between each group (*p* < 0.05). Minimal cytotoxicity was observed against CD19^−^ Jurkat cells, and no statistical significance was found between groups. These results confirmed the expected cytolytic activity of the iCAR19 T cells.

## 3. Discussion

In this study, we incorporated a Tet-on system into the CD19CAR construct to generate the inducible CD19CAR T cells and examined the feasibility of regulating the CAR expression using doxycycline. In the preliminary test, an inducible GFP vector was developed and exhibited dox-induced GFP expression in 293T cells. In subsequent experiments, the iCAR19 T cells showed dox-dependent cell proliferation, cytokine production, CAR expression, and strong CD19-specific cytotoxicity, suggesting that the activity of iCAR19 T cells was successfully controlled by the Tet-on system. Besides, for rapid clinical translation, the produced lentiviral vectors and CAR T cells were GMP-grade, since all manufacture processes were conducted according to the GMP standards.

The striking clinical success of the CAR T-cell therapy in hematologic malignancies and the FDA approval of two CD19CAR T-cell products has attracted huge academic and commercial interest in this promising technology. At the same time, the severe side effects associated with this therapy have also raised wide concern for its safe use [15,16]. Different approaches have been investigated to control the functional activity of CAR T cells. In a few most recent studies, it was reported that tetracycline regulatory systems incorporated in retroviral vectors could effectively control CAR expression and CAR T-cell function in a dox-dose dependent manner without compromising the original anti-tumor efficacy [17,18], consistent with our findings here. Our work here further extended the application of the Tet-inducible system to the third-generation lentiviral vector, which has demonstrated satisfactory safety profile and wide applicability. Compared to the suicide gene systems [19], the inducible gene system offers the possibility to shut down the cytolytic activity of CAR T cells without losing their viability. While research is being conducted to target more diseases like solid tumors using various types of tumor-associated antigens and immune cells [20], this inducible gene system can also be adopted in these new therapies to improve their safety.

Also, we observed a moderate level of basal activation of this Tet-on system, which caused undesired CAR expression and functional leakage of CAR T cells in the uninduced state. The expression leakage was also reported in a previous study utilizing a similar one-vector Tet-on system [18], while no leakage was observed when using a two-vector system [15]. Clearly, different vector designs would influence the basal activation level; however, the exact molecular mechanism has yet to be clarified. From a safety perspective, one-vector system has a better safety profile because it can minimize the risk of oncogene activation due to the integration of vector into the host genome [21].

One limitation in this study is that no selection marker was incorporated in our vector design, thus the transduced CAR T cells were not selected. This is because we intended to minimize the genetic modification to the T cells for easier clinical translation. In addition, selection processes such as FACS could induce great damage and unpredictable changes to T cells. However, the transduction efficiency in our study was found to be suboptimal (~25%). Hence, the anti-tumor activity of our inducible CAR T cells could be greatly enhanced by increasing the purity. Possible solutions to achieve higher transduction efficiency may involve reducing vector size [22] and utilizing better transduction techniques like microfluidic systems [23]. Apart from this, in vivo anti-tumor experiments were not conducted due to limited time and resources. To reduce basal activation and improve transduction efficiency, different vector design of tetracycline regulatory systems and lentiviral transduction techniques will be compared and optimized. Furthermore, in vivo experiments will be conducted to further examine the efficacy of the inducible system.

Another potential problem with this inducible system is the possible adverse effects associated with the inducer and the immune response in clinical use. Prolonged exposure to doxycycline as inducer may cause systemic toxicity as well as increased risk of generating antibiotic-resistant microorganisms in the patients [24]. To increase safety, the minimum dose of doxycycline required to induce sufficient transgene expression and therapeutic efficacy needs to be determined and applied in future in vivo experiments. In addition, development of alternatives to doxycycline that have no antibiotic properties such as the doxycycline metabolite or the tetracycline agonist could be an ideal solution [25]. In addition to the inducer, the potential immunogenicity of the various prokaryotic and viral components in the rtTA fusion protein also raised safety concerns [26]. In a previous clinical study, HSV-TK-modified T cells provoked robust and durable CD8^+^ and CD4^+^ T-cell immune responses towards HSV-TK, resulting in the disappearance of the transferred cells [27]. Since the VP16 domain of the rtTA2S-M2 trans-activator is also derived from the herpes simplex virus and most people have been in contact with this virus, this Tet-inducible system may also elicit immune response against the engineered T cells after infusion [28,29,30]. However, the immunogenicity of the Tet-inducible system may also be influenced by targeted cell types, as immune responses were not detected when they were used in rat brains and dog eyes [31,32]. Hence, further studies need to be conducted to characterize and minimize the potential immunogenicity of the Tet-inducible system on different in vivo models.

In conclusion, GMP-grade inducible CD19CAR T cells were developed in this work. By utilizing a Tet-on system, the activity of the CAR T cells can be controlled in a dox-dependent manner. The incorporation of tetracycline regulatory system into the CAR constructs may be a promising strategy to enhance the safety of CAR T cells.

## 4. Materials and Methods

### 4.1. Cell Lines

All tumor cell lines were obtained from the American Type Culture Collection and cultured in RPMI-1640 medium containing 10% fetal bovine serum (FBS), 0.8 mmol/L l-glutamine, and 1% penicillin–streptomycin. HEK293T cell line (Procell, Hong Kong, China) was cultured in standard DMEM supplemented with 10% FBS, 1% penicillin–streptomycin and 2 mM glutamine.

### 4.2. Vector Constructs

The schematic representation of the lentiviral vectors designed in this study was shown in Figure 1. The Tet-on sequence with SnaBI restriction site on the 5’ end and SalI restriction site on the 3’ end was synthesized on pUC57 vector (Sangon Biotech, Shanghai, China). Then the Tet-on sequence was amplified and cloned into SnaBI-SalI digested pCDH-CMV-MCS-EF1α-copGFP vector (System Biosciences, Palo Alto, CA, USA) to create the pCDH-induce vector. To generate the inducible GFP (iGFP) control vector, GFP sequence was amplified from pCDH-CMV-MCS-EF1α-CopGFP vector with primers carrying SalI digest sites, then cloned into SalI digested pCDH-induce vector. To generate iCAR19 transfer vector, the CD19CAR fragment was synthesized with SalI sites at both ends (Sangon Biotech) and ligated into the SalI digested pCDH-induce vector.

### 4.3. Lentiviral Vector Production

Lentiviral vectors were produced by transfection of HEK293T cells as described in Reference [13]. Briefly, HEK293T cells (7 × 10^6^/plate) were growing in 15-cm dishes for 24 h prior to transfection in DMEM with 10% FBS. The culture medium was exchanged 2 h before transfection (DMEM with 2% FBS). The calcium phosphate precipitation method was used for transient transfection of HEK293T cells. A total of 40 μg of plasmid DNA was used for the transfection of one culture dish, including 12 μg of the envelope plasmid (pMD2.G) encoding VSV-G, 5 μg of the packaging plasmid (pMDLg/pRRE), 3 μg of the plasmid producing Rev regulatory protein (pRSV-Rev), and 20 μg of the transfer vector (iCAR19 and iGFP). After post-transfection culture, all the lentiviral vectors were collected, concentrated, and stored at −80 °C until use.

### 4.4. Lentiviral Vector Quality Testing

#### 4.4.1. Microbiological Tests

Microbiological tests were performed for bacteria, fungi, and mycoplasma using Blood Agar plates, Sabouraud Glucose Agar plates, and LookOut^®^ Mycoplasma PCR Detection Kit, respectively, according to the manufacturers’ protocol (All from Sigma-Aldrich, St. Louis, MI, USA).

#### 4.4.2. Titration

The transducing unit titer (TU/mL) was determined by quantitative PCR (qPCR) as described in Reference [33], with primers targeting the lentivirus WPRE sequence. Human β-actin was used as the reference gene.

#### 4.4.3. Replication-Competent Recombinant Lentivirus

The lentiviral vector stocks were tested for the presence of replication-competent retrovirus by detecting the HIV-1 p24 antigen in the culture medium obtained from vector-transduced cells following several passages in culture, using the Lenti-X™ p24 Rapid Titer Kit (Takara, Kusatsu, Japan) according to the manufacturer’s protocol.

#### 4.4.4. Endotoxin

The endotoxin present in the lentiviral vector stocks was tested using ToxinSensor™ Chromogenic LAL Endotoxin Assay Kit (GenScript, Piscataway, NJ, USA) according to the manufacturer’s protocol.

#### 4.4.5. pH

The pH value of the lentiviral vector stocks was measured and recorded, with a benchtop electronic pH meter (Omega, Biel/Bienne, Switzerland).

### 4.5. HEK293T Cell Transduction

To test our Tet-on system, the iGFP lentiviral vector was used to transduce the 293T cells. Non-transduced cells were used as the control. Cells in 24-well plates with 50–60% confluence were transduced with lentiviral vector at a multiplicity of infection (MOI) of 3 in a total volume of 300 μL of DMEM (10% FBS) supplemented with 8 μg/mL Polybrene. After 24 h, the cells were washed, and fresh medium supplemented with 4 μg/mL doxycycline was added for the induction group. All groups were maintained in culture for another 48 h and observed under fluorescence microscopy (Nikon, Tokyo, Japan) for the expression of GFP marker.

### 4.6. PBMC Transduction

Human peripheral blood mononuclear cells (PBMCs) were transduced with iCAR19 lentiviral vector to produce the iCAR19 T cells. Whole blood was collected from healthy volunteers at Shanghai East Hospital with donors’ written consent. The PBMCs were separated from whole blood, activated with CD3 and CD28 magnetic beads, and cultivated in serum-free medium supplemented with 50 IU/mL interleukin-2 (IL-2). Cells were transduced on day 5 with iCAR19 lentiviral vector at MOI of 3 in the presence of 8 μg/mL polybrene. The non-transduced PBMCs were used as the control. After 24 h, 4 μg/mL doxycycline was added to the culture medium for the induction groups. Cells were harvested after 48 h of induction for subsequent analysis. For the dox discontinuation group, cells were washed and cultured in fresh medium without dox for another 24 h.

### 4.7. Quantitative Real-Time PCR

To analyze the CAR expression level, total RNA from different groups was extracted and reverse transcribed into cDNA. Chimeric antigen receptor expression in mRNA level was evaluated in a StepOnePlus™ Real-Time PCR System (Applied Biosystems, Foster City, CA, USA) using primers targeting CD19 sequence. β-actin mRNA was used as the reference gene. The 2^−∆∆*C*t^ method was used to analyze the relative fold change of gene expression. Each sample was tested in triplicates within one real-time PCR run.

### 4.8. Cell Proliferation and Cytokine Secretion Assays

For proliferation analysis, 1 × 10^6^ iCAR19 T cells with or without dox supplementation (4 μg/mL) were co-cultured with irradiated CD19^+^ LCLs as stimulators at a T cell/LCL ratio of 1:10 in the presence of 50 IU/mL IL-2. The numbers of viable cells were counted at weekly intervals by hematocytometer. For cytokine secretion analysis, iCAR19 T cells were stimulated with irradiated Raji cells at a 5:1 ratio (10^5^ tumor cells) with or without dox supplementation. Supernatants were collected 24 h after stimulation, and concentrations of interleukin-2 (IL-2) and interferon-gamma (IFNγ) were measured by ELISA.

### 4.9. Cytotoxicity Assays

The bioluminescent-based cytotoxicity assay was performed as described in Reference [15]. After 48 h of induction, iCAR19 T cells were incubated with Raji and K562 cells stably transduced with lentiviral vector encoding firefly luciferase (Lentigen Technology, Gaithersburg, MD, USA) at an E:T ratio of 5:1. After incubation for 16 h, luciferase signal produced by surviving target cells was determined by adding SteadyGlo reagent (Promega); % lysis cells = (1 − (signal in treated wells/signal in untreated wells)) × 100%.

Additionally, flow cytometry-based cytotoxicity assay was performed. Raji cells were labeled by carboxyfluorescein succinimidyl ester (CFSE; Thermo Fisher Scientific, Waltham, MA, USA) before coculture with iCAR19 T cells at an effector: target cell ratio (E:T ratio) of 5:1 overnight. The percentage of viable target cells were determined by flow cytometry (AriaII; BD Biosciences, San Jose, CA, USA).

### 4.10. Statistical Analysis

All statistical analysis was performed using GraphPad Prism 7 software with *p* < 0.05 considered to be statistically significant. Difference between groups was evaluated by Student’s *t*-test or one-way ANOVA analysis followed by multiple comparisons of individual groups, where appropriate.

## Figures and Tables

**Table 1:** iCAR19 lentiviral vector quality testing result.

Test	Method	Requirement	Report
Bacteria	Blood agar plates	No growth	No growth
Fungi	Sabouraud glucose agar plates	No growth	No growth
Mycoplasma	PCR detection kit	Negative	Negative
Titration	Real-time PCR	Report TU/mL	1.5 × 10^7^ TU/mL
RCR test	p24 ELISA kit	Negative	Negative
Endotoxin	Chromogenic LAL assay kit	<10 EU/mL	<0.03125 EU/mL
pH	pH meter	6.9–7.8	7.25

## Abbreviations

iCAR19	inducible CD19CAR
CAR	chimeric antigen receptor
CRS	cytokine release syndrome
Dox	doxycycline
TRE	tetracycline-responsive element
TREalb	Tet-responsive promoter for albumin
GMP	Good Manufacturing Practice
FBS	fetal bovine serum
iGFP	inducible GFP
PBMCs	peripheral blood mononuclear cells
IL-2	interleukin-2
IFNγ	interferon-gamma
CFSE	carboxyfluorescein succinimidyl ester
scFv	single-chain variable fragment
